# Prenatal Maternal Docosahexaenoic Acid (DHA) Supplementation and Newborn Anthropometry in India: Findings from DHANI

**DOI:** 10.3390/nu13030730

**Published:** 2021-02-25

**Authors:** Shweta Khandelwal, Dimple Kondal, Monica Chaudhry, Kamal Patil, Mallaiah Kenchaveeraiah Swamy, Gangubai Pujeri, Swati Babu Mane, Yashaswi Kudachi, Ruby Gupta, Usha Ramakrishnan, Aryeh D. Stein, Dorairaj Prabhakaran, Nikhil Tandon

**Affiliations:** 1 Public Health Foundation of India, Gurugram 122003, India; dimple@ccdcindia.org (D.K.); monica.chaudhry@phfi.org (M.C.); ruby.gupta@phfi.org (R.G.); dprabhakaran@phfi.org (D.P.); 2 Centre for Chronic Disease Control, New Delhi 110016, India; 3 Department of Obstetrics and Gynaecology, KAHER’s J. N. Medical College, Belagavi 590010, India; kamalpatil1967@yahoo.co.in (K.P.); mkswamy53@yahoo.co.in (M.K.S.); pujerig@yahoo.in (G.P.); maneswati43@gmail.com (S.B.M.); yash.kudachi@gmail.com (Y.K.); 4 Hubert Department of Global Health, Rollins School of Public Health, Emory University, Atlanta, GA 30322, USA; uramakr@emory.edu (U.R.); aryeh.stein@emory.edu (A.D.S.); 5 All India Institute of Medical Sciences, New Delhi 110016, India; Nikhil_tandon@hotmail.com

**Keywords:** docosahexaenoic acid (DHA), long chain omega-3 fatty acids, maternal supplementation, pregnancy outcomes, anthropometry, birth weight, birth length, head circumference

## Abstract

Long-chain omega-3 fatty acid status during pregnancy may influence newborn anthropometry and duration of gestation. Evidence from high-quality trials from low- and middle-income countries (LMICs) is limited. We conducted a double-blind, randomized, placebo-controlled trial among 957 pregnant women (singleton gestation, 14–20 weeks’ gestation at enrollment) in India to test the effectiveness of 400 mg/day algal docosahexaenoic acid (DHA) compared to placebo provided from enrollment through delivery. Among 3379 women who were screened, 1171 were found eligible; 957 were enrolled and were randomized. The intervention was two microencapsulated algal DHA (200 × 2 = 400 mg/day) or two microencapsulated soy and corn oil placebo tablets to be consumed daily from enrollment (≤20 weeks) through delivery. The primary outcome was newborn anthropometry (birth weight, length, head circumference). Secondary outcomes were gestational age and 1 and 5 min Appearance, Pulse, Grimace, Activity, and Respiration (APGAR) score. The groups (DHA; *n* = 478 and placebo; *n* = 479) were well balanced at baseline. There were 902 live births. Compliance with the intervention was similar across groups (DHA: 88.5%; placebo: 87.1%). There were no significant differences between DHA and placebo groups for birth weight (2750.6 ± 421.5 vs. 2768.2 ± 436.6 g, *p* = 0.54), length (47.3 ± 2.0 vs. 47.5 ± 2.0 cm, *p* = 0.13), or head circumference (33.7 ± 1.4 vs. 33.8 ± 1.4 cm, *p* = 0.15). The mean gestational age at delivery was similar between groups (DHA: 38.8 ± 1.7 placebo: 38.8 ± 1.7 wk, *p* = 0.54) as were APGAR scores at 1 and 5 min. Supplementing mothers through pregnancy with 400 mg/day DHA did not impact the offspring‘s birthweight, length, or head circumference.

## 1. Introduction

Birth weight is a key predictor of the health trajectory of a child [1]. In 2015, the global prevalence of low birth weight (LBW) was recorded to be 14.6%, and 91% of these were from low- and middle-income countries (LMICs), primarily in southern Asia (48%) and sub-Saharan Africa (24%) [2]. LBW and preterm birth are leading causes of neonatal death in LMICs [3]. In addition, LBW is associated with an increased risk of numerous adverse health outcomes in childhood [4,5] and adulthood [6,7]. Women in deprived socio-economic conditions frequently have poor nutrition and consequently deliver infants with LBW [8]. Evidence from several studies, including birth cohorts in Brazil, Guatemala, India, The Philippines, and South Africa [9], shows that poor fetal growth carries a higher risk of chronic diseases related to nutrition later in adult life.

LBW can be the result of preterm birth (PTB) and/or intrauterine growth restriction (IUGR). The underlying causes of both PTB and IUGR are multi-factorial, including infectious diseases, hypertensive disorders, trauma and illness, maternal characteristics, and social determinants. However, the etiologies lead to a common pathway of insufficient uterine–placental perfusion and fetal nutrition [10]. Among the maternal characteristics, maternal nutritional status has been identified as one of the key determinants for LBW in India [11]. Current dietary recommendations for pregnant women emphasize protein, energy, vitamin, and mineral adequacy, but increasing attention is being given to dietary lipids, especially essential fatty acids (EFAs) [12]. Long-chain polyunsaturated fatty acid (LC-PUFA) intake during pregnancy influences both maternal and infant fatty acid status at birth [13], which itself is associated with birth weight and gestational age at birth [14]. A substantial proportion of the Indian population is vegetarian (35%, ranging from 10% to 62% across regions) or observes religious dietary restrictions that can result in multiple nutrient deficiencies [15]. Since the main dietary source of DHA is oily fish, non-supplemented vegetarian diets contain little DHA, and vegan diets contain virtually none. Indian women have low intakes of omega-3 fatty acids—median alpha linolenic acid (ALA), eicosapentanoic acid (EPA) and docosahexaenoic acid (DHA) levels are 560, 3, and 1.1 mg/day during pregnancy, respectively [16]. This is significantly lower than the daily EPA and DHA consumption recommended by the Food and Agriculture Organization (FAO) [17] (2010), for pregnant and lactating women (300 mg per day EPA + DHA, of which 200 mg per day is DHA).

Growing evidence suggests that supplementation during pregnancy with omega-3 fatty acids, especially DHA, may improve birth outcomes. In a prospective cohort study from southern India, women who did not eat fish during the third trimester had a significantly higher risk of LBW (OR: 2.49, *p* = 0.019) when compared to women whose intake was above median, that is, 9.33 g/day (interquartile range: 5.10–15.69) [18]. A review by Makrides and Best [19], documenting the global evidence on epidemiological studies and trials conducted in this area, suggested that N-3 LCPUFA supplementation during pregnancy increased the mean duration of gestation by 2 days; there was also a 40–50% reduction in early preterm birth (<34 weeks’ gestation) [19]. In the United States of America, DHA supplementation resulted in longer gestation duration (2.9 d; *p* = 0.041) and greater birth weight (172 g; *p* = 0.004), length (0.7 cm; *p* = 0.022), and head circumference (HC) (0.5 cm; *p* = 0.012) [20]. Among Mexican women randomized to 400 mg/day of algal DHA or placebo from 18 to 22 weeks of gestation through delivery, the intent-to-treat analysis showed no differences between the placebo and DHA groups in newborn anthropometry, but offspring of supplemented primigravidae were 99.4 g heavier (95% CI, 5.5 to 193.4) and had 0.5 cm larger HC (diff = 95% CI, 0.1 to 0.9) than controls [21]. In the DHA to Optimize Mother Infant Outcome (DOMInO) trial from Australia, women who received fish oil supplements had a lower risk of very preterm birth (1.09% in the DHA group compared to 2.25% in the control group); mean birth weight was 68 g (95% CI, 23–114 g) heavier, and fewer infants had LBW (3.41% vs. 5.27%; 95% CI, 0.44–0.96) [22].

As results have been inconsistent, and little research on this question comes from LMIC contexts where the underlying nutritional status and etiology of LBW may differ, we assessed the impact of maternal DHA supplementation on newborn anthropometry, APGAR score, duration of gestation, and low birth weight among Indian women.

## 2. Materials and Methods

### 2.1. Trial Design and Setting

DHANI (effect of *n*-3 fatty acid (DHA) supplementation during pregnancy on newborn birth weight and gestational age in India) was established as a randomized, double-blinded, placebo-controlled trial to assess the effect of 400 mg/day algal prenatal DHA consumption by healthy Indian women from ≤20 weeks of singleton gestation till delivery on their offspring’s size (weight, length, and head circumference) at birth. The detailed trial protocol has been published elsewhere [23]. DHANI is registered on the CTRI website as CTRI/2013/04/003540 and at clinical trials.gov as NCT01580345. Ethical clearance was obtained from institutional review boards (IRBs) of all participating institutions: Center for Chronic Disease Control (CCDC-IEC_04_2015), Public Health Foundation of India (PHFI) (TRC-IEC-261/15), and Jawaharlal Nehru Medical College (MDC/IECHSR/2016-17/A-85).

### 2.2. Participants and Trial Procedures

The study population was healthy pregnant women, aged 18–35 years with singleton pregnancy under ≤20 weeks of gestation, with no obstetric high-risk conditions, medical complications, or chronic diseases, attending the Department of Obstetrics and Gynecology at the Prabhakar Kore Hospital (PKH) in Belgavi, a largely rural district in Karnataka State, southwest India for antenatal care. Designated project staff approached women, and the consulting obstetrician on site, considering obstetric history and complications, affirmed final eligibility. Consenting eligible women were randomized by project staff to receive either 400 mg/day DHA or a placebo after providing written informed consent using a form in their preferred local language (Kannada, Marathi, or Hindi) and observed by a witness. Information on sociodemographic characteristics, obstetric and medical history, dietary intake (with a pre-piloted semi-quantitative food frequency questionnaire focusing on *n*-3 LC-PUFA-rich Indian foods), anthropometric measurements, a non-fasting blood draw, and vital signs were obtained at enrollment. The women were then given the supplements in the form of coded bottles (each bottle had a 2 week supply) matching the allotted code for the participant. Further supplements were either collected by the women from the study site or were delivered to the women’s homes every fortnight by fieldworkers.

Research staff maintained contact with all women, especially during the last trimester, and visited the woman in the delivery ward within 24 h of delivery to collect data on gestational age at delivery, type of delivery, complications (if any), pregnancy outcome, APGAR (Appearance, Pulse, Grimace, Activity, Respiration) score, newborn anthropometry (weight, length, and head circumference), and maternal and cord blood samples.

### 2.3. Randomization, Masking, and Intervention

The randomization list for 1200 women was generated using a permuted block design (randomly allocating 600 women to DHA or placebo). The assignment code list was placed in a sealed envelope at the beginning of the study and in a secure location at PHFI by a staff member not involved in the trial. Study participants and research staff (including those at the study site) remained blinded to the treatment allocation throughout the duration of fieldwork. After obtaining due approval from the Data Safety Monitoring Board (DSMB) of the study, full analyses were carried out. Unblinding of the treatment group was done only after the generation of the primary tables.

The details of the intervention have been published already [23], but briefly, the intervention was comprised of 635 mg soft gel capsules having either 200 mg/day algal DHA or a placebo (soy/corn oil in a 50:50 ratio), identical in taste and appearance. The active ingredient DHA-S (also known as “DHA algal oil”) is a naturally occurring, microalgal oil derived from *Schizochytrium* sp. (DSM Nutritional Products, Columbia, MD, USA). The sealed capsules had a shelf life of 2 years from the date of manufacture when stored at room temperature (25 °C) and 90 days once the bottle was opened. The women were instructed to store capsules in a cool, dry place and to take two capsules daily, preferably at the same time each day. Supplements were provided for more than two weeks in cases where the woman shared plans to travel. Enrolled women received supplements from the date of randomization through 6 months postpartum; for the present analysis, only supplement intake through delivery was considered.

### 2.4. Outcomes

The primary outcome for the DHANI trial was newborn anthropometry (birth weight, birth length, head circumference). Secondary outcomes included gestational age, APGAR scores at 1 and 5 min, still births, LBW, and preterm. All research staff at the study site were apprised of the data collection methods before the start of the trial and were provided regular refresher training every 6 months. Abstracted data included gestational age, pregnancy outcome (live birth, sex of baby, type of delivery), and APGAR score at 1 min and 5 min. Gestational age at delivery was calculated in weeks by noting the number of days from the last menstrual period (LMP) until delivery. Preterm delivery was defined as delivery after 20 weeks and before 37 completed weeks. Anthropometric data were collected by a trained research assistant within 24 h of delivery. Birth weight was measured to the nearest 10 g by using a portable single-pan digital pediatric weighing scale. Low birth weight was defined as recorded birth weight less than 2500 g. Birth length and head circumference were measured by trained research staff to the nearest 1 mm using a portable anthropometer with a fixed headpiece and a non-stretchable measuring tape, respectively, according to standard procedures. Fetal losses during pregnancy—including miscarriages/abortions and still-births and the APGAR scores were obtained from the hospital records by study personnel on-site, or details were brought by field workers (in case mother went to any other hospital). Stillbirths were defined as fetuses delivered at 20 weeks of gestation or later with no signs of life and recorded as occurring before or during the onset of labor; neonatal deaths were defined as deaths among live-born infants occurring within 28 days after delivery.

### 2.5. Adherence and Follow Up

Subjects were asked to maintain a daily record of their supplement consumption using a form provided by study staff. Weekly calls were made by the research staff to encourage compliance and inquire about general well-being. The used bottles were collected (for pill count) by the field-workers during the fortnightly home visits. The compliance was calculated as the total number of capsules actually consumed, expressed as a percentage of the total number expected to be consumed, which was assessed based on a compliance form filled by the participant and verified by the research staff at all home visits. A sub-sample of venous blood samples collected from the mother at recruitment and delivery was analyzed for DHA levels.

### 2.6. Statistical Analysis

Using data from published literature from another developing country setting [21], we estimated that a sample of 350 mothers per group would have at least 80% power to detect an effect size of 0.20 standard deviation (SD) or greater for the primary outcomes (birth weight and gestational age) at the end of the study, with a significance level of 0.05 for a two-tailed test. A 10% loss to follow-up during pregnancy and 45 as the neonatal mortality rate (NMR) were taken into account. This sample size would also allow us to detect minimum differences in birth weight of 100 g (0.2 SD) between groups with at least 80% power.

Baseline maternal and offspring characteristics were summarized as means and standard deviations or medians and inter-quartile ranges as appropriate, and categorical variables were summarized using proportions.

We used a two-sample *t*-test to compare the differences in mean birth weight, birth length, head circumference, and APGAR score at 1 min and 5 min at delivery between the DHA and placebo groups. We also calculated the *z* score for birth weight, length, and head circumference using standards established by the (International Fetal and Newborn Growth Consortium for the 21st century (INTERGROWTH-21) Project [24] and compared the difference in *z* score between DHA and placebo group using two-sample *t*-tests. The differences in proportion for preterm birth and LBW between the DHA and placebo groups were compared using the two-proportion z-test. The analysis was done using the intent to treat (ITT) principle.

We conducted several pre-specified subgroup analyses to estimate the treatment effects within different categories of maternal age (18–20, 21–25, 26–30, 31–35 years), body mass index (BMI) at enrollment (<18.5 kg/m^2^; 18.5–23.0 kg/m^2^; 23.0–27.5 kg/m^2^; and 27.5 kg/m^2^) as per Asian cut-offs [25], gravidity (multi-gravida, primi gravida), gestational age at delivery (<37, ≥37 weeks), compliance (<80.0%, ≥80.0%), vegetarian diet (yes, no), and child sex (male, female). The *p*-value for heterogeneity was calculated by including the interaction term between the characteristic of interest and treatment group in the linear regression model. The significance of within-subgroup treatment effects was adjusted for multiplicity for multiple subgroup analyses using the Bonferroni criterion, i.e., by dividing the overall significance level by the total number of subgroup analyses performed. For sensitivity analysis, we compared the baseline characteristics between the final study sample and those who were lost to follow-up. *p* values <0.05 were considered to be statistically significant. All statistical analysis was done using STATA 16.0 version (College Station, TX, USA) and R 3.6.2 software (Free Software Foundation, Inc., Boston, MA, USA).

## 3. Results

### 3.1. Trial Population

**Figure 1:**
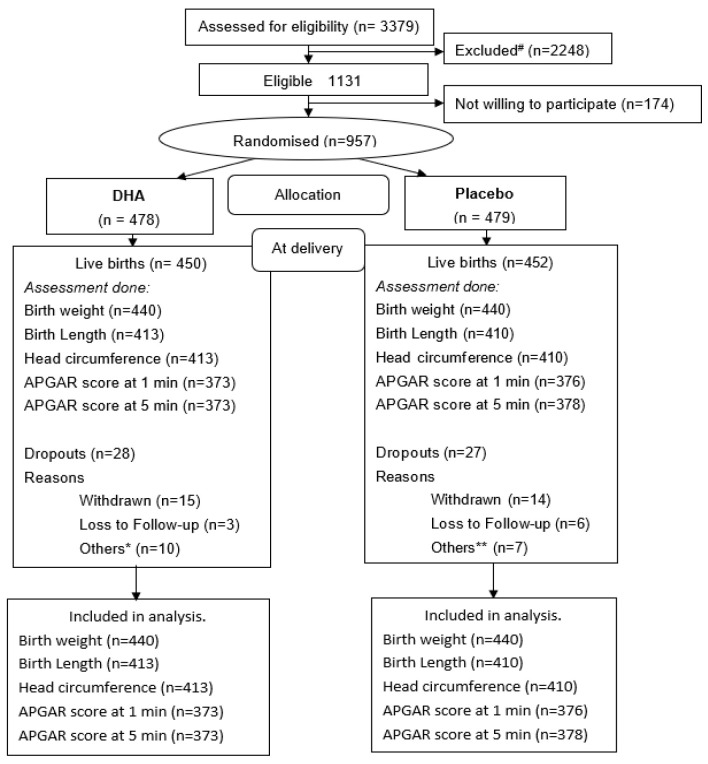
Consort ^#^ reasons for exclusion: gestational diabetes (*n* = 69); Hb < 7 g% (*n* = 46); gestational age > 20 weeks (*n* = 673); high risk pregnancies (*n* = 118); chronic conditions (*n* = 246); under any other trial (*n* = 4); delivery plan other than PK (*n* = 835); missing/wrong contact information (*n* = 257). * Others included abortion (*n* = 1); abruptio placenta (*n* = 1); fresh still birth (*n* = 4); macerated still birth (*n* = 3); neonatal death (*n* = 1) in DHA group. ** Others included fresh still birth (*n* = 4); macerated still birth (*n* = 2); medical termination (*n* = 1) in placebo group.

A total of 3379 women were screened, and 1131 were found to be eligible. Among these, 957 mothers provided informed consent and were randomly assigned to receive DHA (*n* = 478) or placebo (*n* = 479) (Figure 1).

Overall, the mean (SD) age of the mothers was 23.5 (3.6) years, and gestational age (median (interquartile interval)) at enrolment was 15.0 (12.0, 18.0) weeks. A total of 79% of the women had completed at least secondary school and 23% of the women were employed. About 12% of the women reported monthly household income more than Rs 20,000 (285 USD taking 1 USD = 70 INR). Baseline characteristics of the enrolled women were similar between DHA and placebo groups (Table 1). The details have been published elsewhere [23]. In addition, there was no difference in baseline characteristics between those who were followed up till delivery and those who were not (Supplementary Materials Table S1).

The two groups did not differ in estimated intake of energy or any macronutrient at baseline (Supplementary Table S2 and Table 2). The mean DHA levels at baseline and delivery by birth weight (<2500 g; ≥2500 g), length (<50 cm; ≥50 cm), and head circumference (<34 cm; ≥34 cm) are shown in Table 3. The mean DHA levels at baseline did not differ overall between the two groups. A significant change was observed in the mean DHA values at delivery, being higher in the DHA group as compared to placebo, both overall and when subdivided by birth weight, length, head circumference, and gestational age.

Supplementary Materials Table S3 shows the mean change in DHA levels from baseline to delivery by treatment group. There is an increase in mean DHA level in both the groups from baseline to delivery (DHA 1.20 (0.98, 1.43), *p* = <0.001; placebo 0.24 (0.11, 0.36), *p* = 0.0002).

There were 450 (94.1%) and 452 (94.3%) live births in the DHA (*n* = 478) and placebo (*n* = 479) groups, respectively. Compliance was high in both groups (DHA: 88.5% and placebo: 87.1%). There were 230 (52.3%) and 235 (53.4%) male children in the DHA and placebo groups, respectively, and percentages were calculated based on 440 (DHA) and 440 (placebo) analyzed neonates.

### 3.2. Outcomes

Table 3 shows birth outcomes for all live births according to the treatment group. There were no significant differences between DHA and placebo groups for mean birth weight (2750.6 ± 421.5 vs. 2768.2 ± 436.6 g, *p* = 0.54), birth length (47.3 ± 2.0 vs. 47.5 ± 2.0 cm, *p* = 0.13), or head circumference (33.7 ± 1.4 vs. 33.8 ± 1.4 cm, *p* = 0.15). The APGAR scores at 1 min and 5 min were similar between the groups. We did not find any significant difference between DHA and placebo groups in *z* scores for birth weight, length, and head circumference.

Gestational age at delivery was similar between DHA and placebo groups (DHA vs. placebo: 38.8 ±1.7 vs. 38.8 ± 1.7 wk, *p* = 0.54). The prevalence of preterm birth and low birth weight did not differ significantly between the groups. Unfortunately, the causes of preterm birth and the number of intra uterine growth retardation (IUGR) cases were not collected.

### 3.3. Sub-Group Analysis

**Figure 2:**
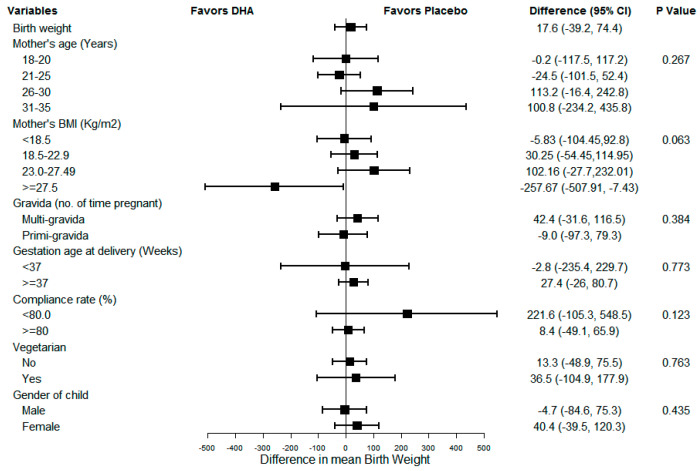
Sub-group analysis for newborn birthweight.

**Figure 3:**
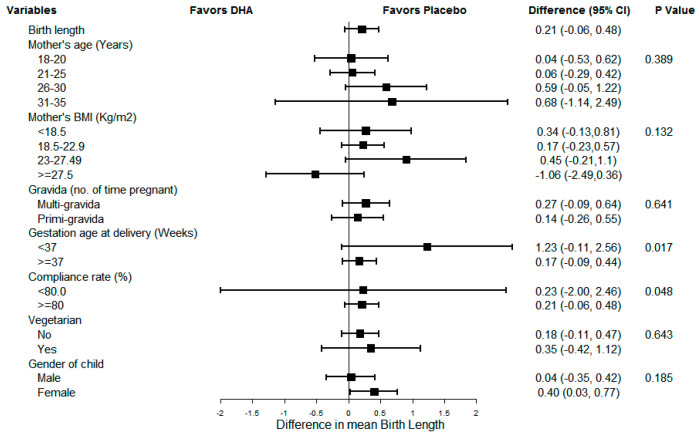
Sub-group analysis for newborn birth length.

**Figure 4:**
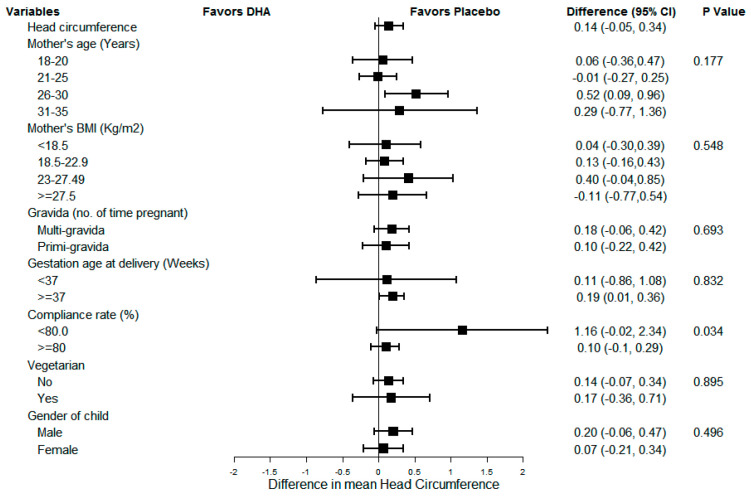
Sub-group analysis for newborn head circumference.

Figure 2, Figure 3 and Figure 4 show the results of sub-group analyses for birth weight, birth length, and head circumference, respectively. The effect of DHA on the birth size (i.e., weight, length, and head circumference) did not differ across any of the subgroups examined (*p* = 0.007, *p*-value adjusted for multiplicity using Bonferroni correction). Similarly, there was no evidence of differences by compliance, the gender of the child, or preterm status.

## 4. Discussion

In this study, maternal supplementation with 400 mg/day DHA in the second half of pregnancy did not affect the weight, length, or head circumference of the offspring at birth. While this was in contrast to findings from some high-income settings [14], it concurs with other studies from relatively comparable settings [21].

Although mechanistic pathways linking maternal polyunsaturated fatty acid (PUFA), especially DHA status with gestational length, are poorly delineated, prenatal DHA supplementation has been shown to enhance the gestation duration in some studies [26]. This longer gestation duration with fish oil that contains EPA as well as DHA may be due to an alteration in the balance of prostaglandins derived from EPA and arachidonic acid [27]. A high proportion of omega-6 to omega-3 FAs can contribute to increased pro-inflammatory eicosanoids (i.e., prostaglandin E2 (PGE2) and prostaglandin F2 (PGF2)) production. These metabolites have been shown to be linked with the initiation of labor and premature labor. Including more EPA in the diet may lead to a reduction in the production of pro-inflammatory eicosanoids and expanded production of prostacyclin (PGI2), which may promote myometrial relaxation. Omega-3 LC-PUFA, especially DHA, downregulates production of prostaglandins PGE2 and PGF2 and may thus inhibit the process of parturition. This has been postulated to be associated with increased gestation duration and the accretion of intrauterine LC-PUFA [28]. Longer gestation indeed also influences newborn anthropometry positively, and thus DHA was shown to also confer small benefits on newborn anthropometry because of its impact on gestation duration. However, our trial did not find any such benefit.

A recent 2018 Cochrane review [29] looking at the impact of omega 3 fatty acids (including both DHA and EPA) concludes that omega 3 LCPUFA reduces the incidence of preterm birth <37 weeks and early preterm birth <34 weeks in women receiving omega-3 LCPUFA compared with no omega-3s. Thus, in our study the supplementation of DHA without other fractions like EPA may not have been able to result in an effect on gestation length. This review of high-quality evidence from 15 trials with 8449 participants also noted that there was a reduced risk LBW (15.6% versus 14%; RR 0.90, 95% CI 0.82 to 0.99) [29]. Increased birthweight due to prenatal DHA supplementation has been observed in only primiparous women [30]. The authors suggest that since primiparous women were, on average, younger than multiparous women, their own body stores of DHA are not well established and available to the fetus and infant [30]. Ramakrishnan et al., from the same cohort, showed that the offspring of primigravid women who received DHA were heavier at birth than the offspring of primigravid women who received placebo (difference, 99.4 g; 95% CI, 5.5 to 193.4) and had larger head circumferences (difference, 0.5 cm; 95% CI, 0.1 to 0.9 cm) [21]. In the current study, however, the woman’s parity did not affect the effect of DHA on the newborn’s birth weight, length, or head circumference.

Key strengths of this study are the strong study design combined with high retention rates and compliance (verified by the rise in erythrocyte DHA levels).

Another parameter which is often of interest is the timing of initiation of supplementation during pregnancy. The other salient trials [14,19] initiated DHA supplementation during mid pregnancy (14.5 weeks and 19 weeks median, respectively). Similarly, in our trial, DHA supplementation started between 12 and 18 weeks of pregnancy, with a median value of 15 weeks. Nevertheless, we did not observe any impact of DHA on the outcomes, unlike the two other trials.

The complexity of multiple other factors apart from DHA in affecting birth size needs to be recognized. Factors like the maternal diet at multiple time points during pregnancy, family support, stress levels [31], and the consumption of other important micronutrients like iron and zinc that were not assessed may have influenced birth size [32]. Further, we do not have data on the single nucleotide polymorphisms (SNPs) in the fatty acid desaturase (FADS) gene that has been known to affect the activity of the enzymes that convert PUFAs into their long-chain active form and may determine who benefits from supplementation [33,34]. Future large-scale trials taking into account all these factors are warranted.

## 5. Conclusions

In summary, no beneficial effects of prenatal supplementation of Indian women with DHA from mid-pregnancy through delivery on newborn anthropometry were observed.

## Figures and Tables

**Table 1:** Maternal anthropometrics and DHA level according to treatment group at randomization.

Variable	DHA (*n* = 478)	Placebo (*n* = 479)
Maternal age (year), mean ± SD	23.5 ± 3.5	23.6 ± 3.7
Gestational age at enrollment (weeks), median (p25, p75)	15.0 (12.0, 18.0)	14.0 (12.0, 18.0)
Weight (kg), mean ± SD	48.9 ± 9.0	48.9 ± 8.5
Height (cm), mean ± SD	154.1 ± 5.6	153.9 ± 5.7
Body mass index (kg/m^2^), mean ± SD	20.5 ± 3.5	20.7 ± 3.6
MUAC (cm), mean ± SD	24.3 ± 3.0	24.3 ± 3.1
Hb (g%), mean ± SD	11.1 ± 1.3	11.2 ± 1.3
DHA (mol% of fatty acid) *, mean ± SD	0.86 ± 0.78	0.88 ± 0.71

MUAC: mid upper arm circumference; Hb: hemoglobin; DHA: docosahexaenoic acid; * *n* = 258 (DHA); *n* = 224 (placebo).

**Table 2:** Mean DHA (mol% of fatty acid) levels in RBC phospholipids.

DHA Levels	DHA	Placebo	Mean Difference * (95% CI)	*p*-Value
	*n*, Mean ± SD, Median (p25, p75)	*n*, Mean ± SD, Median (p25, p75)		
Overall				
DHA at baseline	*n* = 256, 0.86 ± 0.78, 0.56 (0.31, 1.20)	*n* = 224, 0.88 ± 0.71, 0.55 (0.37, 1.28)	0.02 (−0.11, 0.15)	0.770
DHA at delivery	*n* = 269, 2.03 ± 1.76, 1.41 (0.61, 2.99)	*n* = 242, 1.12 ± 0.86, 0.83 (0.42, 1.72)	−0.91 (−1.16, −0.67)	<0.001
Birth Weight < 2500 g				
DHA at baseline	*n* = 63, 0.96 ± 0.89, 0.59 (0.39, 1.41)	*n* = 47, 0.74 ± 0.69, 0.46 (0.37, 0.95)	−0.22 (−0.53, 0.09)	0.170
DHA at delivery	*n* = 67, 2.00 ± 1.81, 1.39 (0.63, 2.79)	*n* = 50, 1.17 ± 0.80, 0.96 (0.48, 1.74)	−0.83 (−1.37, −0.29)	0.003
Birth Weight ≥ 2500 g				
DHA at baseline	*n* = 193, 0.83 ± 0.75, 0.53 (0.3, 1.11)	*n* = 177, 0.92 ± 0.71, 0.59 (0.37, 1.33)	0.09 (−0.06, 0.24)	0.221
DHA at delivery	*n* = 202, 2.04 ± 1.74, 1.43 (0.6, 3.17)	*n* = 192, 1.10± 0.88, 0.78 (0.4, 1.7)	−0.94 (−1.22, −0.66)	<0.001
Gestation Age < 37 Weeks				
DHA at baseline	*n* = 18, 1.1 ± 0.74, 0.79 (0.61, 1.55)	*n* = 19, 0.55 ± 0.37, 0.41 (0.37, 0.66)	−0.55 (−0.93, −0.16)	0.007
DHA at delivery	*n* = 17, 2.24 ± 1.81, 1.72 (0.96, 2.97)	*n* = 19, 0.99 ± 0.72, 0.62 (0.41, 1.72)	1.25 (−2.16, −0.33)	0.009
Gestation Age ≥ 37 Weeks				
DHA at baseline	*n* = 238,0.84 ± 0.79, 0.53 (0.31, 1.14)	*n* = 205,0.91 ± 0.72, 0.59 (0.37, 1.3)	−0.07 (−0.07, 0.21)	0.333
DHA at delivery	*n* = 252, 2.02 ± 1.76, 1.4 (0.59, 3.08)	*n* = 223, 1.13 ± 0.87, 0.83 (0.42, 1.72)	−0.89 (−1.15, −0.64)	<0.001
Birth Length < 50 cm				
DHA at baseline	*n* = 234, 0.89 ± 0.81, 0.58 (0.32, 1.33)	*n* = 194, 0.85 ± 0.71, 0.52 (0.34, 1.23)	−0.04 (0.1, −0.33)	0.556
DHA at delivery	*n* = 245, 2.04 ± 1.77, 1.39 (0.62, 3.17)	*n* = 213, 1.1 ± 0.86, 0.78 (0.42, 1.72)	−0.94 (-0.68, −0.33)	<0.0001
Birth Length ≥ 50 cm				
DHA at baseline	*n* = 21, 0.51 ± 0.33, 0.44 (0.28, 0.59)	*n* = 30, 1.11 ± 0.64, 1 (0.62, 1.47)	0.6 (0.91, −0.33)	0.0003
DHA at delivery	*n* = 24, 1.97 ± 1.7, 1.72 (0.58, 2.45)	*n* = 29, 1.28 ± 0.87, 1.14 (0.54, 1.73)	−0.69 (0.04, −0.33)	0.063
Head Circumference < 34 cm				
DHA at baseline	N = 123, 0.95 ± 0.89, 0.59 (0.34, 1.36)	N = 98, 0.82 ± 0.66, 0.53 (0.39, 1.23)	−0.12 (0.09, −0.33)	0.258
DHA at delivery	n=132, 2.16 ± 1.81, 1.49 (0.73, 2.98)	N = 106, 1.16 ± 0.89, 0.93 (0.45, 1.74)	−1.00 (−0.62, −0.33)	<0.0001
Head Circumference ≥ 34 cm				
DHA at baseline	*n* = 132, 0.78 ± 0.67, 0.52 (0.29, 0.97)	*n* = 126, 0.93 ± 0.74, 0.57 (0.34, 1.4)	0.15 (0.32, −0.33)	0.0916
DHA at delivery	*n* = 137,1.91 ± 1.7, 1.19 (0.55, 3.17)	*n* = 136, 1.08 ± 0.84, 0.78 (0.41, 1.7)	−0.83 (−0.51, −0.33)	<0.0001

DHA levels were analyzed only in a subset of women; data are presented as mean ± standard deviation, median (p25, p75); DHA: docosahexaenoic acid; *p*-Value calculated using unpaired *t*-test; * difference = placebo minus DHA.

**Table 3:** Birth outcomes for all live births according to treatment group.

Birth Outcomes	DHA		Placebo		Mean Difference ^§^ (95% CI)	*p*-Value
	*n*	Mean ± SD/n (%)	*n*	Mean ± SD/n (%)		
Gestational age at delivery (weeks)	440	38.8 ± 1.7	440	38.8 ± 1.7	0.07 (−0.16, 0.30)	0.54
Preterm birth (gestation < 37 week) ^†^	440	28 (6.4%)	440	33 (7.5%)	0.01 (−0.02, 0.04)^‡^	0.52
Newborn Anthropometry						
Birth weight (grams)	440	2750.6 ± 421.5	440	2768.2 ± 436.6	17.6 (−39.2, 74.4)	0.54
Low birth weight (<2500 g) ^†^	440	105 (23.9%)	440	99 (22.5%)	−0.01 (−0.07, 0.04) ^‡^	0.63
Birth length (cm)	413	47.3 ± 2.0	410	47.5 ± 2.0	0.21 (−0.06, 0.48)	0.13
Birth head circumference (cm)	413	33.7 ± 1.4	410	33.8 ± 1.4	0.14 (−0.05, 0.34)	0.15
Apgar score at 1 min	372	6.9 ± 0.8	376	6.9 ± 0.8	0.01 (−0.11, 0.13)	0.91
Apgar score at 5 min	373	8.0 ± 0.7	378	8.0 ± 0.7	0.03 (−0.07, 0.12)	0.60
Size for Gestational Age and Sex According to Standardized Measures ^¶^						
Birth weight for gestational age *z* score	440	−0.97 ± 0.98	440	−0.95 ± 0.95	0.03 (−0.1, 0.16)	0.67
Birth length for gestational age *z* score	413	−0.84 ± 1.04	410	−0.73 ± 1.12	0.11 (−0.03, 0.26)	0.13
Birth head circumference for gestational age *z* score.	413	0.09 ± 1.05	410	0.20 ± 0.97	0.11 (−0.03, 0.25)	0.11
Small for gestational age *^,@^	440	172 (39.1%)	440	172 (39.1%)	na	na

^†^*n* (%); ^‡^ difference in proportions reported; ^§^ difference = (placebo − DHA); difference in mean values reported using two-sample *t*-test. Difference in proportions reported using proportion test; ^¶^ standards are based on those established by the INTERGROWTH-21st (International Fetal and Newborn Growth Consortium for the 21st century) Project [23]. ^@^: Infants considered to be small for gestational age had a weight-for-age *z* score that was below the 10th percentile according to neonatal standards established by the INTERGROWTH-21st Project. Na: not applicable.

## Data Availability

The request for accessing de-identified data plus data dictionary will be put forth for approval by the trial’s mentoring and advisory committee (MAC). Interested researchers may submit a proposal with a valid reason or justification, e.g., meta-analysis, etc.

## Supplementary Materials

The following are available online at https://www.mdpi.com/2072-6643/13/3/730/s1: Table S1: Comparison of baseline characteristics comparing women who continued to participate in the study through delivery and those who did not, Table S2: Dietary data on subsample at randomization (*n* = 278); Table S3: Mean change in DHA levels from baseline to delivery.
