# Supplementary material for: Prenatal Maternal Docosahexaenoic Acid (DHA) Supplementation and Newborn Anthropometry in India: Findings from DHANI
Source: Nutrients. 2021 Feb 25;13(3):730. doi: 10.3390/nu13030730 (PMC7996222; doi:10.3390/nu13030730)
Supplement: Supplementary file 1 [file nutrients-13-00730-s001.pdf]

Supplementary Tables

Table S1. Comparison of baseline characteristics between women who continued to participate in the study through delivery and those who did not.

Variable(s)	Randomized and Continued through Delivery (*N* = 880)	Randomized but Did Not Continue through Delivery (*N* = 55)	*p*-Value
Maternal age (year), mean ± SD	23.5 ± 3.6	23.7 (3.9)	0.68
Gestational age at enrolment (weeks), median (p25, p75)	15.0 (12.0, 18.0)	14.0 (11.0, 17.0)	0.11
Primigravida, *n* (%)	349 (39.7%)	26 (47.3%)	0.26
Education, *n* (%)			
College graduated and above	158 (18.0%)	7 (12.7%)	0.41
High school/Secondary	695 (79.0%)	45 (81.8%)	
Employed, n (%)	213 (24.2%)	7 (12.7%)	0.052
Household monthly income (INR), n (%) (Rs > 20,000)	102 (11.6%)	4 (7.3%)	0.48
Treatment assignment, n (%)			
DHA	440 (50.0%)	28 (50.9%)	0.90
Placebo	440 (50.0%)	27 (49.1%)	

Table S2. Dietary data on subsample at randomization (*n* = 278).

Macronutrient fractions	DHA (*N* = 140)	Placebo (*N* = 138)	*p*-Value
Energy (Kcal), mean ± SD	1358.2 ± 431.0	1391.4 ± 370.9	0.49
Energy (Kcal), median (p25, p75)	1231.6 (1066.3, 1682.2)	1347.3 (1088.4, 1629.6)	0.22
Protein (g), mean ± SD	48.9 ± 13.7	49.2 ± 12.7	0.84
Protein (g), median (p25, p75)	45.2 (39.7, 59.1)	48.7 (40.6, 56.8)	0.64
Fat (g), mean ± SD	38.8 ± 16.6	38.2 ± 13.1	0.73
Fat (g), median (p25, p75)	35.9 (28.2, 45.4)	36.3 (28.8, 45.0)	0.92
Carbohydrates (g), mean ± SD	202.3 ± 75.4	212.4 ± 71.4	0.25
Carbohydrates (g), median (p25, p75)	178.0 (156.2, 247.3)	198.1 (163.4, 254.3)	0.14

Kcal: kilocalories; g: grams; data are presented as mean ± standard deviation or median (p25, p75); (p25, p75): interquartile interval. *p*-Value for difference in mean values calculated using two-sample *t*-test. *p*-Value for difference in median values calculated using Wilcoxon rank sum test.

Table 3. Mean change in DHA levels from baseline to delivery.

DHA (Mol% of Fatty Acid)	Baseline	Delivery	Mean Difference *	*p*-Value
DHA group, mean (SD)	0.87 (0.79)	2.08 (1.75)	1.20 (0.98, 1.43)	<0.001
median (p25, p75)	0.57 (0.32, 1.22)	1.52 (0.62, 3.17)		
Placebo group, mean (SD)	0.89 (0.71)	1.13 (0.86)	0.24 (0.11, −0.36)	0.0002
median (p25, p75)	0.56 (0.36, 1.29)	0.83 (0.41, 1.73)		

* Difference: delivery minus baseline; DHA (*n* = 250) placebo (*n* = 216). Mean difference and *p* value calculated using paired *t*-test.
